# Body fatness during childhood and adolescence and incidence of breast cancer in premenopausal women: a prospective cohort study

**DOI:** 10.1186/bcr998

**Published:** 2005-02-18

**Authors:** Heather J Baer, Graham A Colditz, Bernard Rosner, Karin B Michels, Janet W Rich-Edwards, David J Hunter, Walter C Willett

**Affiliations:** 1 Channing Laboratory, Department of Medicine, Brigham and Women's Hospital, Boston, Massachusetts, USA; 2 Department of Epidemiology, Harvard School of Public Health, Boston, Massachusetts, USA; 3 Harvard Center for Cancer Prevention, Boston, Massachusetts, USA; 4 Department of Biostatistics, Harvard School of Public Health, Boston, Massachusetts, USA; 5 Obstetrics and Gynecology Epidemiology Center, Brigham and Women's Hospital and Harvard Medical School, Boston, Massachusetts, USA; 6 Department of Ambulatory Care and Prevention, Harvard Pilgrim Health Care and Harvard Medical School, Boston, Massachusetts, USA; 7 Department of Nutrition, Harvard School of Public Health, Boston, Massachusetts, USA

## Abstract

**Introduction:**

Body mass index (BMI) during adulthood is inversely related to the incidence of premenopausal breast cancer, but the role of body fatness earlier in life is less clear. We examined prospectively the relation between body fatness during childhood and adolescence and the incidence of breast cancer in premenopausal women.

**Methods:**

Participants were 109,267 premenopausal women in the Nurses' Health Study II who recalled their body fatness at ages 5, 10 and 20 years using a validated 9-level figure drawing. Over 12 years of follow up, 1318 incident cases of breast cancer were identified. Cox proportional hazards regression was used to compute relative risks (RRs) and 95% confidence intervals (CIs) for body fatness at each age and for average childhood (ages 5–10 years) and adolescent (ages 10–20 years) fatness.

**Results:**

Body fatness at each age was inversely associated with premenopausal breast cancer incidence; the multivariate RRs were 0.48 (95% CI 0.35–0.55) and 0.57 (95% CI 0.39–0.83) for the most overweight compared with the most lean in childhood and adolescence, respectively (*P *for trend < 0.0001). The association for childhood body fatness was only slightly attenuated after adjustment for later BMI, with a multivariate RR of 0.52 (95% CI 0.38–0.71) for the most overweight compared with the most lean (*P *for trend = 0.001). Adjustment for menstrual cycle characteristics had little impact on the association.

**Conclusion:**

Greater body fatness during childhood and adolescence is associated with reduced incidence of premenopausal breast cancer, independent of adult BMI and menstrual cycle characteristics.

## Introduction

Body mass index (BMI) during adulthood is related to breast cancer incidence, although the direction of the association varies by menopausal status. More overweight women have a lower risk of breast cancer before menopause but a higher risk after menopause [1,2]. In premenopausal women, greater adiposity may increase the frequency of anovulatory menstrual cycles, thus reducing exposure to ovarian hormones [3]. In contrast, greater adiposity in postmenopausal women increases both estrogen levels and breast cancer risk, which is probably due to the conversion of androgens to estrone in adipose tissue [3].

Despite the consistency of evidence for adult BMI, few studies have examined the role of body fatness during childhood and early adolescence, and results have been inconclusive. The majority have been case-control studies in which body fatness at young ages was recalled after diagnosis of breast cancer. Some [4-7] but not all [8-11] of these studies have shown inverse associations between body fatness at young ages and breast cancer risk. In a study that linked census records for residents of Hawaii to tumor registry data [12], a strong inverse association was seen between prospectively recorded body fatness from ages 10–14 years and incidence of premenopausal breast cancer; a study conducted in Finland that used height and weight measurements for ages 7–15 years from school health records [13] yielded similar findings. However, limited information on potential confounders was available in these studies. In an analysis that combined retrospective and prospective data within the earlier Nurses' Health Study [14], inverse associations were observed for recalled body fatness at ages 5, 10, and 20 years in relation to incidence of both premenopausal and postmenopausal breast cancer, with the strongest inverse association of these three for body fatness at age 10 years. A prospective cohort study conducted in Norway and Sweden [15] found inverse associations of perceived body shape at age 7 years and BMI at age 18 years with incidence of premenopausal breast cancer, but these associations were attenuated and no longer significant when adjusted for BMI at enrollment. A recent Danish study that used information from school health records [16] also found a modest inverse association between BMI at age 14 years and breast cancer risk, although no data on adult BMI were available.

A variety of evidence points to the importance of early life factors in the etiology of breast cancer. Studies of mammary gland development in rats have shown that developing breast tissue may be most vulnerable to carcinogens before the first birth, when undifferentiated cells are undergoing rapid proliferation [17,18]. Epidemiologic studies conducted in humans and biomathematical models also suggest that the years between menarche and first birth may be a critical time period for breast carcinogenesis [19]. In light of these findings, it is plausible that body fatness at young ages could influence risk of breast cancer later in life.

We examined prospectively the relation between body fatness during childhood and adolescence and incidence of breast cancer among premenopausal women in the Nurses' Health Study II (NHS II). In addition, we investigated whether this relation is independent of characteristics of the menstrual cycle and BMI during adulthood.

## Materials and methods

### Study design and population

The NHS II is a prospective cohort study that began in 1989, when 116,671 female registered nurses between the ages of 25 and 42 years completed a mailed, self-administered questionnaire about their health behaviors, lifestyle factors, and medical histories. Follow-up questionnaires have been sent to participants every 2 years to obtain updated information on risk factors and disease diagnoses, and the response rate for each biennial questionnaire has been greater than 90%. Deaths are reported by family members and the postal service, and regular searches of the computerized National Death Index are also conducted [20]. This analysis includes the 109,267 premenopausal women who provided information on their body fatness at ages 5, 10, and 20 years on the initial questionnaire in 1989 and who had no history of cancer (other than nonmelanoma skin cancer). The study was approved by human research committees at the Harvard School of Public Health and Brigham and Women's Hospital.

### Ascertainment of breast cancer cases

On each of the biennial questionnaires between 1989 and 2001, participants were asked whether they had been diagnosed with breast cancer during the previous 2 years or since the last questionnaire they returned. Study physicians then confirmed the self-reported diagnoses by reviewing participants' medical records and/or pathology reports.

A total of 1318 cases of breast cancer (1044 invasive, 274 *in situ*) were reported and subsequently confirmed among eligible participants during 12 years of follow-up, from 1989 to 1 June 2001. Because epidemiologic studies have generally shown similar risk factors for *in situ *and invasive breast cancer [21-23], both are included in our primary analyses, although secondary analyses in which the outcome was restricted to invasive disease were also conducted.

### Assessment of body fatness at young ages

**Figure 1:**
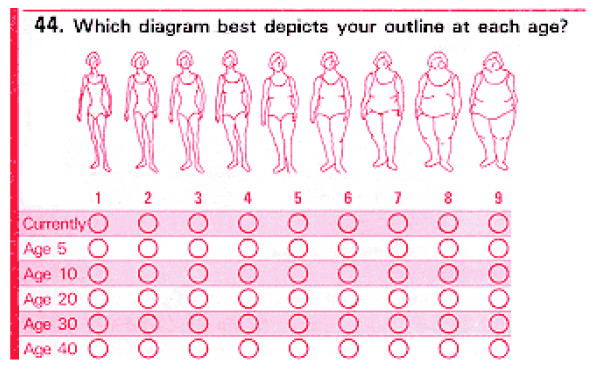
Figure drawing 24 used to assess body fatness at different ages among Nurses' Health Study II participants.

In 1989, NHS II participants recalled their body fatness at ages 5, 10, and 20 years using a 9-level figure drawing (Fig. 1) originally developed by Stunkard and colleagues [24]. Participants who did not report their body fatness at one or more of these ages were excluded. To obtain estimates of childhood and adolescent body fatness, we averaged each participant's figures at ages 5 and 10 years (childhood) and at ages 10 and 20 years (adolescence); the goal of this approach was to reduce the effects of random error in the assessment of body fatness. Changes in body fatness between ages 5 and 10 years, between ages 10 and 20 years, and between ages 5 and 20 years were also calculated by subtracting each participant's figure (levels 1 through 9) at the younger age from that at the older age.

Must and colleagues [25] evaluated the validity of remote recall of body fatness among 181 participants in the Third Harvard Growth Study, a longitudinal study of physical and mental growth in children that was conducted between 1922 and 1935 in the Boston area. Height and weight were measured as part of annual examinations during childhood and adolescence and were used to calculate BMI in kg/m^2^. In 1988 and 1989, when participants were between ages 71 and 76 years, they were interviewed again and asked to recall their body fatness at ages 5, 10, 15, and 20 years, using the same 9-level figure drawing as that on the 1989 NHS II questionnaire. Pearson correlations between recalled body fatness and BMI at approximately the same ages were 0.60 for age 5 years, 0.70 for age 10 years, 0.75 for age 15 years, and 0.66 for age 20 years. Other studies have yielded similar findings [26-29], indicating that these figure drawings can provide a reasonably accurate assessment of body fatness at young ages.

Among participants in the Third Harvard Growth Study, which provides the best available 'gold standard' for the interpretation of the figure drawing, the median values for BMI from measured height and weight at age 15 years according to recalled figure at age 15 years were 19, 19.5, 24, 25, 25.5, 30, and 32 kg/m^2 ^for levels 1 through 7, respectively [25]; no participants reported being greater than level 7. In the present study, the median values for BMI at age 18 years (based on self-reported data) according to recalled figure at age 20 years were 18.1 for level 1, 19.1 for level 2, 20.5 for level 3, 22.3 for level 4, 24.9 for level 5, 28.2 for level 6, 32.0 for level 7, 34.7 for level 8, and 37.8 kg/m^2 ^for level 9.

### Assessment of other risk factors

Information on other established and hypothesized breast cancer risk factors was collected at various points during the course of the study. Age, menopausal status, reproductive history, oral contraceptive use, smoking status, and diagnosis of benign breast disease were reported at baseline in 1989 and updated on each of the biennial questionnaires. History of breast cancer in a first-degree relative (mother or sister) was reported in 1989 and updated in 1997. Recent alcohol consumption was assessed on the initial questionnaire in 1989 and again in 1991, 1995, and 1999 from semiquantitative food frequency questionnaires, which also evaluated other dietary factors. Participation in physical activity was evaluated in 1989, 1991, and 1997. Height, alcohol consumption between ages 15 and 17 years and ages 18 and 22 years, and strenuous physical activity during high school and between ages 18 and 22 years were assessed in 1989. Other early life factors such as birthweight and being breastfed as an infant were reported on the 1991 questionnaire. A subset of participants (*n *= 43,317) provided information on dietary factors during adolescence by completing a supplementary questionnaire on high school diet in 1998.

Weight at age 18 years was reported in 1989, and current weight was reported on each of the biennial questionnaires; these were used with height to calculate BMI at age 18 years and current BMI. Age at menarche, time from menarche until onset of regular menstrual cycles, and cycle regularity and length during high school and between ages 18 and 22 years were reported on the 1989 questionnaire, and recent menstrual cycle characteristics were reported in 1993.

### Analysis

Participants contributed person-time from the return date of the 1989 questionnaire until the report of breast cancer or other cancer (except nonmelanoma skin cancer), menopause, death, or the end of follow-up on 1 June 2001. Cases and person-time were assigned to the appropriate level of body fatness at each age and other risk factors. For time-varying covariates such as oral contraceptive use and parity, person-time was re-assigned every 2 years.

Breast cancer incidence rates for each level of body fatness at ages 5, 10, and 20 years, and during childhood and adolescence were calculated as the number of breast cancer cases divided by the total number of person-years at each level. Few participants recalled their body fatness as greater than level 5 at ages 5 and 10 years; for example, for body fatness at age 10 years, 9.1% of participants recalled their figure as level 5, 2.6% recalled their figure as level 6, 0.4% recalled their figure as level 7, and only 0.1% recalled their figure as either level 8 or level 9. For this reason, figures 5 through 9 were combined into a single category for most analyses, and similar categories were created for childhood and adolescent body fatness. Relative risks (RRs) were calculated by taking the ratio of the incidence rate for each level compared with the lowest level, which was used as the referent category.

Cox proportional hazards regression was used to estimate multivariate RRs and 95% confidence intervals (CIs) for body fatness at young ages while adjusting for age, time period, and other covariates. Body fatness at each age was examined in a separate Cox model, because recalled figures at these ages are highly correlated with one another. Indicator variables were used to obtain RRs for levels of body fatness at each age, and tests for linear trend were conducted by entering body fatness at each age into a Cox model as an ordinal variable with values 1 through 5. Changes in body fatness between ages 5, 10, and 20 years were also examined in Cox models, using the categories decreased, no change, increased 1 level, and increased 2 or more levels, with no change being the referent category.

Because BMI during adulthood and menstrual cycle characteristics could be intermediate factors on the pathway from body fatness at young ages to breast cancer, these were considered separately from other covariates. To determine whether the association between body fatness at young ages and breast cancer is independent of later BMI, we adjusted for later BMI using several different variables, included in separate models: BMI at age 18 years, current BMI (updated every 2 years), and the cumulative average of BMI at age 18 years and all subsequent BMI reports up until the current time period. These variables were each divided into five categories (not including those who were missing) and included as continuous terms by assigning the median value of each category. Childhood body fatness and body fatness at age 20 years were also included in the same multivariate model, to examine the estimates mutually adjusted for one another. Height was included as a continuous term in all multivariate models, because it is positively associated with breast cancer incidence [1] and may reflect nutritional status during childhood [30].

To determine whether body fatness at young ages may have an impact on breast cancer risk by altering characteristics of the menstrual cycle, we first adjusted for age at menarche and years from menarche until the onset of regular menstrual cycles, because greater body fatness at young ages has been associated with earlier menarche but longer time until the establishment of regular cycles [31]. In addition, we considered adjustment for regularity and length of cycles during several different time periods: high school, ages 18–22 years, and recent (reported only once in 1993). With the exception of age at menarche, which was modeled as a continuous variable, indicator variables were used to represent categories of menstrual cycle characteristics.

After conducting these analyses in the full sample, we then restricted them to certain subgroups of participants: women who had had at least one screening mammography during the follow-up period (*n *= 89,129; 1223 cases), women who had no reports of infertility (*n *= 82,892; 991 cases), and women who had no history of irregular menstrual cycles (*n *= 51,909; 666 cases). This was done to explore further whether differential screening or anovulation could account for associations between body fatness at young ages and premenopausal breast cancer incidence. The 1044 invasive cases were also examined in separate models and further stratified by tumor size (574 cases <2 cm; 321 cases ≥ 2 cm) and hormone receptor status (604 estrogen receptor positive cases; 252 estrogen receptor negative cases; 571 progesterone receptor positive cases; 267 estrogen receptor negative cases) when this information was available.

We also examined whether associations for childhood fatness varied according to family history of breast cancer (yes, no), age at menarche (<12 years, ≥ 12 years), parity (nulliparous, parous), oral contraceptive use (never or past use <4 years, past use ≥ 4 years, or current use), birthweight (<7 lb, ≥ 7 lb), BMI at age 18 years (<22 kg/m^2^, ≥ 22 kg/m^2^), or current BMI (<25 kg/m^2^, ≥ 25 kg/m^2^). Separate models were constructed within each level of these factors to obtain stratum-specific estimates, and interaction terms were created by multiplying childhood body fatness as an ordinal variable by the level of each potential modifier. Wald tests were then used to evaluate whether the trends for body fatness were significantly different according to these factors.

## Results

The 109,267 premenopausal women in the analytic cohort contributed a total of 1,044,691 person-years of follow-up. Fatness levels at each of these ages were positively correlated with one another and with BMI at age 18 years and in 1989, although the correlations decreased with time. For example, the Spearman correlation between figures at ages 5 and 10 years was 0.81, whereas the correlation between figure at age 5 years and BMI in 1989 was only 0.25. Body fatness at young ages was also associated with other characteristics (Table 1). Women who were fatter at age 10 years were heavier at birth, had earlier menarche, had higher caloric intake and were less likely to have participated in strenuous physical activity during adolescence, and in later life they were more likely to be nulliparous and to smoke. A greater proportion of participants in both the lowest and highest categories of body fatness at age 10 years reported 3 or more years from menarche until the onset of regular menstrual cycles, whereas those in the highest category were slightly more likely to have had irregular or long cycles between ages 18 and 22 years.

Body fatness at ages 5, 10, and 20 years were each inversely associated with premenopausal breast cancer risk (Table 2). The multivariate RRs for the most overweight (figure level ≥ 5) compared with the most lean (figure level 1) were 0.57 (95% CI 0.43–0.75; *P *for trend = 0.001) for age 5 years, 0.61 (95% CI 0.49–0.76; *P *for trend < 0.0001) for age 10 years, and 0.70 (95% CI 0.52–0.94; *P *for trend < 0.0001) for age 20 years, after adjustment for age and time period, birthweight, height, recent alcohol consumption, parity and age at first birth, recency and duration of oral contraceptive use, history of benign breast disease, and family history of breast cancer. The associations of average childhood and adolescent body fatness with breast cancer incidence were slightly stronger than the associations at individual ages (Table 3), with multivariate RRs of 0.48 (95% CI 0.35–0.65) and 0.57 (95% CI 0.39–0.83) for the most overweight compared with the most lean in childhood and adolescence, respectively (*P *for trend < 0.0001). Additional adjustment for adolescent intakes of animal fat, vegetable fat, and vitamin E [32,33] among those participants who completed the high school diet questionnaire, physical activity during adolescence and adulthood, and recent smoking did not materially change the RRs.

Increases in body fatness during childhood and adolescence were also inversely associated with breast cancer risk (Table 3). Compared with participants who stayed at the same level from age 5 to age 20 years, the multivariate RR for those who increased 2 or more levels was 0.86 (95% CI 0.74–1.02). This association became stronger after adjustment for body fatness at age 5 years, with a multivariate RR of 0.77 (95% CI 0.65–0.91) for those who increased 2 or more levels. Similar patterns were observed for changes in body fatness from ages 5 to 10 years and from ages 10 to 20 years.

We then evaluated whether the inverse association for childhood body fatness was independent of later BMI and menstrual cycle characteristics (Table 4). When average childhood body fatness was adjusted for the cumulative average of BMI at age 18 years and subsequent BMI, the association was only slightly attenuated; the multivariate RR was 0.52 (95% CI 0.38–0.71) for the most overweight compared with the most lean (*P *for trend = 0.001). The results were similar when childhood body fatness was adjusted for BMI at age 18 years and current BMI separately. Adjustment for menstrual cycle characteristics had virtually no impact on the association for childhood body fatness. Furthermore, when the analyses were restricted to participants who reported having regular menstrual cycles during both adolescence and adulthood, and to participants with no reports of infertility, the multivariate RRs for childhood body fatness were almost identical to those in the full sample (data not shown).

In separate models in which BMI at age 18 years and current BMI were adjusted for childhood body fatness, each remained inversely related to breast cancer incidence, although the associations were attenuated and less strong than the association for childhood body fatness. The multivariate RRs for the highest versus the lowest categories of BMI at age 18 years and current BMI, adjusted for childhood body fatness, were 0.84 (95% CI 0.66–1.07) and 0.86 (95% CI 0.71–1.04), respectively. When average childhood body fatness and body fatness at age 20 years were included in the same model as indicator variables, the multivariate RR for the most overweight compared with the most lean in childhood was 0.56 (95% CI 0.40–0.78; *P *for trend = 0.03), whereas the multivariate RR for the most overweight compared with the most lean at age 20 years was 0.82 (95% CI 0.59–1.14; *P *for trend = 0.02).

The associations for childhood and adolescent body fatness were slightly stronger in the analyses including only invasive cases. The multivariate RR for the most overweight compared with the most lean during childhood was 0.45 (95% CI 0.31–0.64; *P *for trend < 0.0001) and during adolescence was 0.52 (95% CI 0.33–0.80; *P *for trend < 0.0001). The decreased risk was apparent both for small and large tumors and for hormone receptor positive and negative tumors (data not shown), although some of the estimates were imprecise because of small numbers of cases. The associations were also very similar among the subgroup of women who had had at least one screening mammography during the follow-up period (data not shown).

The observed associations for childhood body fatness did not differ appreciably by family history of breast cancer, age at menarche, parity, oral contraceptive use, or birthweight (data not shown). The inverse trend across categories of childhood body fatness was somewhat more apparent among those who were heavy than among those who were lean at age 18 years, and the test for interaction was marginally significant (χ^2 ^_1 _= 3.68; *P *= 0.06), although the multivariate RRs for the most overweight compared with the most lean in childhood were very similar in both categories of BMI at age 18 years (RR = 0.42 among those with BMI <22 kg/m^2 ^at age 18 years, *P *for trend = 0.08; and RR = 0.49 among those with BMI ≥ 22 kg/m^2 ^at age 18 years, *P *for trend = 0.001). In contrast, the association for childhood body fatness did not differ according to current BMI (data not shown).

## Discussion

In this prospective study we observed a significant inverse association between body fatness during childhood and adolescence and incidence of breast cancer in premenopausal women, with approximately 50% lower risk for the most overweight compared with the most lean in childhood. The magnitude of the decrease in risk was greater for childhood body fatness than for body fatness at older ages. The inverse association was independent of later BMI and menstrual cycle characteristics, suggesting that body fatness at young ages may influence breast cancer risk through other biologic pathways.

Our findings are consistent with the results of some other studies that have examined this relationship. Le Marchand and colleagues [12] linked prospectively recorded information on height and weight from census data for over 38,000 women to the Hawaii Tumor Registry, from which 607 cases of breast cancer were identified. In that study BMI at ages 5–9 years, 10–14 years, and 20–24 years were each inversely associated with breast cancer incidence, but the strongest association was for BMI from ages 10–14 years, with an odds ratio of 0.51 for the highest versus the lowest tertile. A second prospective study of 3447 women born at the University Hospital of Helsinki [13] obtained anthropometric measurements from birth and school health records and linked this information to the National Hospital Discharge Registry and the Cause of Death registry, identifying 177 incident cases of breast cancer. BMI at ages 7–15 years were inversely associated with breast cancer risk, with a RR of 0.83 for each 1 kg/m^2 ^increase in BMI at age 7 years. Both studies, however, lacked information on other important breast cancer risk factors that might confound these associations. In a prospective cohort study conducted in Norway and Sweden [15], the investigators observed inverse associations of perceived body shape at age 7 years and BMI at age 18 years with incidence of premenopausal breast cancer, with approximately 30% decreased risk for those who were fat or very fat at age 7 years compared with those who were average. In contrast to our findings, the association was strongest for adult BMI at enrollment, and the associations for perceived body shape at age 7 years and BMI at age 18 years were no longer significant after this adjustment. However, only 733 cases were included in that study, which could explain the lack of significance. A recent record linkage study conducted in Denmark that included over 3000 breast cancer cases [16] also observed a modest inverse association for BMI at age 14 years, based on information from school health records, but data on adult BMI were not available.

Other epidemiologic studies have assessed body fatness at young ages through recall. Most of these utilized a case-control design in which women with breast cancer and cancer-free controls were asked to categorize their relative weight compared with other girls at specific ages. The majority found inverse associations for body fatness during the childhood and teenage years, observing a 30–50% decrease in risk for those who reported being heavier or much heavier compared with those who recalled being thin or average size [4,5,7,10]. Several others [8,9,11], however, have not found such inverse associations.

Two previous studies have assessed the relationship between body fatness at young ages and breast cancer risk using the same 9-level figure drawing as in our study. In a large Swedish population-based case-control study of 3345 cases of invasive breast cancer and 3454 controls between ages 50 and 74 years [6], body fatness at ages 7 and 18 years were both inversely associated with postmenopausal breast cancer risk, with a RR of 0.38 for figure level 7 or greater versus level 4 at age 7 years. In a largely retrospective analysis within the earlier Nurses' Health Study [14], body fatness at ages 5, 10, and 20 years were each inversely associated with risk of premenopausal breast cancer, with the strongest association for age 10 years. When body fatness at these ages were mutually adjusted for one another, the RR for figure level 5 or greater compared with level 1 was 0.60 for body fatness at age 10 years. A similar pattern was also observed for postmenopausal breast cancer.

Ours is one of the first prospective studies to examine the relation between childhood body fatness and breast cancer incidence, and we were able to control for a broad range of factors, both in early life and adulthood, that were not available in earlier studies. In addition, unlike most previous studies, we adjusted for later BMI using several different variables, including the cumulative average of BMI at age 18 years and all subsequent BMI reports to obtain the best long-term measure. Even with this adjustment, the inverse associations for body fatness during childhood and adolescence remained strong and statistically significant, suggesting that greater body fatness at early stages of life, perhaps even before puberty, may lower breast cancer risk. In addition, in the analyses stratified by current BMI, greater childhood body fatness was associated with reduced risk of breast cancer among both lean and heavy women, which indicates that greater childhood body fatness may confer a lasting protective effect. The biologic mechanisms that would explain this, however, are not well understood. One theory postulates that more overweight girls may experience slower pubertal growth and sexual maturation, despite their earlier menarche [6,14]. In the Harvard Longitudinal Study of Child Health and Development [34], leaner body mass at age 10 years was predictive of more rapid adolescent growth, and in the Nurses' Health Study [14] adolescents in the highest two quintiles of estimated growth rate had nearly 50% increased risk of premenopausal breast cancer. Rapid adolescent growth may increase breast cancer risk by increasing levels of growth hormones and epithelial proliferation in the breast or by decreasing the amount of time for repair of DNA damage [19].

The effect of body fatness at young ages may also be mediated through hormonal pathways. Obesity in pre-adolescent and adolescent girls is associated with higher basal insulin levels [35,36], which can impair oocyte maturation and stimulate androgen production in the ovary [31,37]. Hyperinsulinemia is also associated with decreased plasma levels of sex hormone binding globulin, leading to increases in free (unbound) testosterone and estradiol, and the aromatization of excess androgen to estrogen in adipose tissue may also increase estrogen levels [38]. Greater waist:hip ratio has been associated with higher serum concentrations of testosterone and estradiol in prepubertal and pubertal girls in some studies [31,39] but not all studies [40,41]. High levels of androgens in adolescent girls are associated with metabolic features of polycystic ovary syndrome [42], greater frequency of anovulatory cycles [37], and reduced fertility later in life [43]. In a previous study conducted among participants in this cohort [44], higher BMI at age 18 years was associated with increased risk of irregular and long menstrual cycles between ages 18 and 22 years as well as increased risk of ovulatory infertility in adulthood [44], and greater body fatness at age 10 years was also associated with moderately increased risk of menstrual cycle irregularities and nulliparity in the present analysis after adjustment for other factors. In addition, menstrual cycle regularity and length were related to breast cancer risk among NHS II participants during the first few years of follow up [45]. However, the observed associations for body fatness at young ages in the present study were nearly identical among participants with no history of irregular menstrual cycles or infertility, suggesting that these are not intermediate factors and that other mechanisms may be involved.

High levels of sex hormones in overweight prepubertal and pubertal girls may also have a more direct protective effect on breast tissue. Several experiments have shown that neonatal, prepubertal, or pubertal administration of estrogen, prolactin, progesterone, or testosterone in rats leads to differentiation of cells of the mammary gland as well as a substantial reduction in the incidence of mammary tumors following exposure to chemical carcinogens [46-50]. Hence, some have recently hypothesized that the timing of exposure to estrogens and other hormones may determine their effects on breast tissue [51,52]. Hilakivi-Clarke [51] has suggested that early estrogen exposure may reduce breast cancer risk by increasing the expression of tumor suppressor genes such as *BRCA1*, inducing differentiation of immature breast cells into more mature ductal structures in addition to stimulating epithelial growth. Higher levels of estrogens may be protective in the breasts of young girls, which are less likely to contain malignant cells, but harmful in older women, whose breasts are more likely to have acquired transformed cells.

Of course, alternative explanations for our findings cannot be ruled out entirely. Although two well designed validation studies [25,29] demonstrated that long-term recall of body fatness using this figure drawing has high correlations with BMI at the same ages, no participants in either of those studies recalled their figure as greater than level 7 at young ages; hence, the accuracy of recall at the highest levels of body fatness could not be assessed. Furthermore, the validation studies showed that women who were obese had a greater tendency to underestimate their body fatness at young ages than those who were lean, which could exaggerate the observed association for less extreme levels of body fatness. However, this would not explain the overall association, and the decreasing trend that we observed in age at menarche across all levels of body fatness at age 10 years is strong evidence of the validity of our assessment. We also repeated the analyses using the middle category of body fatness at each age and during childhood and adolescence as the referent group, to evaluate whether the observed inverse association could possibly be explained by higher risk among participants who were extremely lean at young ages. When we did this, we still observed significantly lower risk for the most overweight compared with the middle category. For example, for average childhood body fatness, the multivariate RR for the most overweight compared with the middle category (level 2.5–3) was 0.53 (95% CI 0.39–0.72), whereas the multivariate RR for the most lean was 1.11 (95% CI 0.95–1.31); this argues against elevated risk among the most lean as the major explanation for our findings. Other unmeasured factors, especially those during early life and childhood, could also confound the associations for body fatness at young ages, although a confounder would have to be very strong to account for an association of this magnitude.

Detection bias is another possibility because women who are obese as adults may be less likely to get regular screening mammograms [53], which could delay or reduce the chance of detection. In this population, however, the probability of having a screening mammography was not appreciably related to childhood body fatness or current BMI among women in several age groups. For example, among women ages 50–54 years in 1999, 69.2% of those who were figure level 1 at age 10 years reported having had a screening mammogram within the preceding 2 years, as compared with 71.8% of those who were figure level 5 or greater at age 10 years. These percentages were similar when examined according to current BMI in 1999 among women ages 50–54 years, although a slightly greater proportion of those in the intermediate category of BMI (23–24.9 kg/m^2^) reported having had a recent screening mammogram compared with those in either of the two extreme categories (74.4% compared with 69.6% for those with BMI <21 kg/m^2 ^and 69.0% for those with BMI ≥ 30 kg/m^2^). We still observed a strong inverse association between early body fatness and breast cancer risk among women who reported having at least one screening mammography. In addition, if easier detection in lean women were the main explanation for our findings, we would have expected the association to become weaker when *in situ *cases were excluded, which is not what occurred. The results stratified by tumor size showed an inverse association for both large and small tumors, also arguing against a detection bias.

## Conclusion

These data indicate that greater body fatness during childhood and adolescence may reduce the incidence of premenopausal breast cancer, independently of adult BMI and menstrual cycle characteristics, and that this association may be stronger than the association for BMI at later ages. These findings should be interpreted cautiously, given that greater adiposity in adolescence has numerous adverse long-term health consequences [54] and that obesity in postmenopausal women increases breast cancer risk [3]. However, they could help to elucidate the biologic mechanisms that are involved in the etiology of breast cancer. In light of the strength of our findings and their consistency with those of previous studies, identifying causal pathways that would help explain this inverse association should be a priority for future research.

## Abbreviations

BMI = body mass index; CI = confidence interval; RR = relative risk.

## Competing interests

The author(s) declare that they have no competing interests.

## Figures and Tables

**Table 1:** Characteristics of 109,267 premenopausal Nurses' Health Study II participants in 1989 according to body fatness at age 10 years

Characteristic	Figure at age 10 years				
	
	1	2	3	4	≥ 5
Number participants (%)	20,554 (18.8)	33,512 (30.7)	24,666 (22.6)	17,274 (15.8)	13,261 (12.1)

Mean					

Age (years)	34.6	34.0	34.1	34.3	34.7
Total caloric intake in high school (kilocalories)^a^	2737	2746	2745	2746	2781
Animal fat intake in high school (% energy)^a^	26.0	25.7	25.8	25.9	26.2
Vegetable fat intake in high school (% energy)^a^	14.4	14.7	14.8	15.0	15.0
Age at menarche (years)	12.8	12.6	12.3	12.1	12.0
Height (inches)	64.9	64.9	64.8	64.8	65.0
BMI at age 18 years (kg/m^2^)	19.3	20.2	21.5	22.9	24.3
Current BMI (kg/m^2^)	22.0	22.7	24.4	26.1	27.1
Age at first birth (years)^b^	25.4	25.5	25.6	25.6	25.4
Parity^b^	2.1	2.1	2.1	2.1	2.0

Percentage					

Birthweight ≥ 8.5 lb	8.3	9.4	11.7	12.9	13.7
≥ 3 years from menarche until regular menstrual cycles	19.5	18.5	17.7	17.9	19.6
Irregular menstrual cycles/no periods ages 18–22 years	9.7	9.0	8.8	10.2	11.2
Menstrual cycle length ≥ 40 days ages 18–22 years	7.1	7.1	7.3	8.2	8.6
Strenuous activity 10–12 months/year in high school	28.7	28.0	26.2	21.4	17.6
BMI at age 18 years ≥ 25 kg/m^2^	1.8	3.1	8.9	19.5	32.2
Current BMI ≥ 30 kg/m^2^	3.0	4.9	11.2	20.2	24.6
Nulliparous	29.6	29.2	29.3	31.3	35.0
Current oral contraceptive user	13.8	14.0	12.6	12.7	11.6
Current smoker	13.1	11.5	12.3	14.2	18.6
Alcohol consumption ≥ 10 g/day	9.7	9.0	9.1	9.1	9.9
First-degree family history of breast cancer	5.9	6.0	5.7	5.9	6.3
History of benign breast disease	30.3	28.1	27.6	27.5	28.0

All means and percentages refer to the 1989 time period unless otherwise noted. Participants who had a diagnosis of cancer (other than nonmelanoma skin cancer) and those for whom body fatness data were missing at ages 5, 10, or 20 years were excluded. ^a^Among participants who completed the high school diet questionnaire in 1998. ^b^Among participants who reported that they were parous in 1989. BMI, body mass index.

**Table 2:** Relative risks of breast cancer by body fatness at ages 5, 10, and 20 years, and during childhood and adolescence among 109,267 premenopausal Nurses' Health Study II participants (1989–2001)

	Cases^a ^(*n *= 1318)	Person-years	Age-adjusted RR (95% CI)	Multivariate RR (95% CI)^b^
Figure at age 5 years				

1	350	252,779	1.00 (ref.)	1.00 (ref.)
2	431	335,974	0.97 (0.84–1.11)	0.96 (0.83–1.11)
3	304	251,625	0.89 (0.76–1.04)	0.88 (0.76–1.03)
4	173	133,574	0.94 (0.78–1.13)	0.93 (0.77–1.12)
≥ 5	60	70,740	0.59 (0.45–0.78)	0.57 (0.43–0.75)
*P *for trend^c^			0.002	0.001

Figure at age 10 years				

1	277	191,720	1.00 (ref.)	1.00 (ref.)
2	423	323,197	0.95 (0.82–1.11)	0.96 (0.82–1.11)
3	307	239,185	0.92 (0.79–1.09)	0.93 (0.79–1.10)
4	197	165,957	0.84 (0.70–1.01)	0.84 (0.70–1.01)
≥ 5	114	124,633	0.62 (0.50–0.77)	0.61 (0.49–0.76)
*P *for trend^c^			<0.0001	<0.0001

Figure at age 20 years				

1	73	44,399	1.00 (ref.)	1.00 (ref.)
2	414	271,292	1.04 (0.81–1.33)	1.05 (0.82–1.35)
3	474	397,651	0.85 (0.67–1.09)	0.87 (0.68–1.11)
4	247	219,136	0.81 (0.62–1.05)	0.81 (0.63–1.06)
≥ 5	110	112,213	0.71 (0.53–0.95)	0.70 (0.52–0.94)
*P *for trend^c^			<0.0001	<0.0001

Average childhood figure (ages 5–10 years)				

1	262	177,512	1.00 (ref.)	1.00 (ref.)
1.5–2	416	322,714	0.92 (0.79–1.08)	0.93 (0.79–1.08)
2.5–3	339	265,111	0.90 (0.77–1.06)	0.90 (0.76–1.06)
3.5–4.5	253	215,175	0.82 (0.69–0.97)	0.81 (0.68–0.96)
≥ 5	48	64,180	0.49 (0.36–0.67)	0.48 (0.35–0.65)
*P *for trend^c^			<0.0001	<0.0001

Average adolescent figure (ages 10–20 years)				

1	56	34,780	1.00 (ref.)	1.00 (ref.)
1.5–2	398	267,086	1.07 (0.81–1.41)	1.08 (0.82–1.43)
2.5–3	449	366,516	0.92 (0.69–1.21)	0.93 (0.71–1.24)
3.5–4.5	363	312,070	0.85 (0.64–1.13)	0.86 (0.65–1.14)
≥ 5	52	64,240	0.58 (0.40–0.85)	0.57 (0.39–0.83)
*P *for trend^c^			<0.0001	<0.0001

^a^Including both invasive and *in situ *cases. ^b^Adjusted for age (months), time period (6 periods), birthweight (<5.5, 5.5–6.9, 7–8.4, 8.5–9.9, ≥ 10 lb), height (inches), recent alcohol consumption (0, 0.1–1.4, 1.5–4.9, 5.0–9.9, ≥ 10 g/day), parity and age at first birth (nulliparous, 1–2 pregnancies with age at first birth <25 years, 1–2 pregnancies with age at first birth 25–29 years, 1–2 pregnancies with age at first birth ≥ 30 years, ≥ 3 pregnancies with age at first birth <25 years, ≥ 3 pregnancies with age at first birth 25–29 years, ≥ 3 pregnancies with age at first birth ≥ 30 years), oral contraceptive use (never, past use <4 years, past use ≥ 4 years, current use <4 years, current use ≥ 4 years), history of benign breast disease (yes, no), and first-degree family history of breast cancer (yes, no). ^c^Wald test of coefficient for body fatness modeled as an ordinal variable. CI, confidence interval; RR, relative risk.

**Table 3:** Relative risks of breast cancer by changes in body fatness between ages 5, 10, and 20 years among 109,267 premenopausal Nurses' Health Study II participants (1989–2001)

	Cases^a ^(*n *= 1318)	Person-years	Age-adjusted RR (95% CI)	Multivariate RR (95% CI)^b^	Multivariate RR + starting figure^c^
Change from ages 5 to 10 years					

Decreased	76	59,972	0.97 (0.77–1.23)	0.94 (0.74–1.19)	1.04 (0.82–1.32)
No change	950	720,994	1.00 (ref.)	1.00 (ref.)	1.00 (ref.)
Increased 1 level	218	194,725	0.87 (0.75–1.01)	0.88 (0.76–1.02)	0.88 (0.76–1.02)
Increased 2 or more levels	74	69,001	0.79 (0.62–1.00)	0.79 (0.62–1.00)	0.77 (0.61–0.98)

Change from ages 10 to 20 years					

Decreased	223	184,978	0.86 (0.74–1.01)	0.86 (0.74–1.01)	1.06 (0.89–1.27)
No change	497	353,987	1.00 (ref.)	1.00 (ref.)	1.00 (ref.)
Increased 1 level	463	388,462	0.92 (0.81–1.04)	0.92 (0.81–1.05)	0.85 (0.74–0.97)
Increased 2 or more levels	135	117,264	0.93 (0.77–1.12)	0.92 (0.76–1.11)	0.82 (0.67–0.99)

Change from ages 5 to 20 years					

Decreased	172	135,479	0.91 (0.76–1.08)	0.90 (0.75–1.07)	1.06 (0.88–1.29)
No change	449	321,121	1.00 (ref.)	1.00 (ref.)	1.00 (ref.)
Increased 1 level	474	384,088	0.94 (0.83–1.08)	0.95 (0.83–1.08)	0.88 (0.77–1.01)
Increased 2 or more levels	223	204,003	0.87 (0.74–1.02)	0.86 (0.74–1.02)	0.77 (0.65–0.91)

^a^Including both invasive and *in situ *cases. ^b^Adjusted for age (months), time period (6 periods), birthweight (<5.5, 5.5–6.9, 7–8.4, 8.5–9.9, ≥ 10 lb), height (inches), recent alcohol consumption (0, 0.1–1.4, 1.5–4.9, 5.0–9.9, ≥ 10 g/day), parity and age at first birth (nulliparous, 1–2 pregnancies with age at first birth <25 years, 1–2 pregnancies with age at first birth 25–29 years, 1–2 pregnancies with age at first birth ≥ 30 years, ≥ 3 pregnancies with age at first birth <25 years, ≥ 3 pregnancies with age at first birth 25–29 years, ≥ 3 pregnancies with age at first birth ≥ 30 years), oral contraceptive use (never, past use <4 years, past use ≥ 4 years, current use <4 years, current use ≥ 4 years), history of benign breast disease (yes, no), and first-degree family history of breast cancer (yes, no). ^c^Adjusted for same factors as above, plus figure at age 5 years (for change from ages 5 to 10 years and from ages 5 to 20 years) or at age 10 years (for change from ages 10 to 20 years), each modeled as an ordinal variable. CI, confidence interval; RR, relative risk.

**Table 4:** Relative risks for breast cancer by average childhood body fatness, with adjustment for later body mass index and menstrual cycle characteristics, among 109,267 premenopausal Nurses' Health Study II participants (1989–2001)

	Average childhood figure (ages 5–10 years)					*P *for trend^d^
		
	1	1.5–2	2.5–3	3.5–4.5	≥ 5	
Multivariate RR (95% CI)^a^	1.00	0.93 (0.79–1.08)	0.90 (0.76–1.06)	0.81 (0.68–0.96)	0.48 (0.35–0.65)	<0.0001

Multivariate RR + later BMI^b^						

BMI at age 18 years	1.00	0.95 (0.81–1.11)	0.95 (0.80–1.12)	0.87 (0.72–1.05)	0.53 (0.38–0.73)	0.004
Current BMI	1.00	0.93 (0.80–1.09)	0.92 (0.78–1.08)	0.84 (0.70–1.01)	0.51 (0.37–0.69)	0.0003
Cumulatively averaged BMI	1.00	0.94 (0.81–1.10)	0.93 (0.79–1.10)	0.86 (0.72–1.03)	0.52 (0.38–0.71)	0.001

Multivariate RR + age at menarche and menstrual cycle characteristics^c^						

Age at menarche	1.00	0.93 (0.80–1.09)	0.89 (0.76–1.05)	0.80 (0.67–0.95)	0.47 (0.34–0.64)	<0.0001
Age at menarche, time until regular cycles	1.00	0.93 (0.80–1.09)	0.89 (0.76–1.05)	0.80 (0.67–0.95)	0.47 (0.34–0.64)	<0.0001
Age at menarche, regularity/length of cycles, ages 18–22 years	1.00	0.93 (0.80–1.09)	0.89 (0.76–1.05)	0.79 (0.67–0.95)	0.47 (0.34–0.64)	<0.0001

^a^Adjusted for age (months), time period (6 periods), birthweight (<5.5, 5.5–6.9, 7–8.4, 8.5–9.9, ≥ 10 lb), height (inches), recent alcohol consumption (0, 0.1–1.4, 1.5–4.9, 5.0–9.9, ≥ 10 g/day), parity and age at first birth (nulliparous, 1–2 pregnancies with age at first birth <25 years, 1–2 pregnancies with age at first birth 25–29 years, 1–2 pregnancies with age at first birth ≥ 30 years, ≥ 3 pregnancies with age at first birth <25 years, ≥ 3 pregnancies with age at first birth 25–29 years, ≥ 3 pregnancies with age at first birth ≥ 30 years), oral contraceptive use (never, past use <4 years, past use ≥ 4 years, current use <4 years, current use ≥ 4 years), history of benign breast disease (yes, no), and first-degree family history of breast cancer (yes, no). ^b^Adjusted for same factors as above, plus later body mass index (BMI) variables individually, each modeled as medians of the categories. Cumulatively-averaged BMI is the cumulative average of BMI at age 18 years and all subsequent BMI reports up until the current time period. ^c^Adjusted for same factors as above (not including later BMI), plus menstrual cycle characteristics. Age at menarche modeled as continuous. Time until onset of regular cycles (<1 year, 1–2 years, ≥ 3 years, never) and cycle regularity and length from ages 18–22 years (regular <26 days, regular 26–31 days, regular ≥ 32 days, irregular <26 days, irregular 26–31 days, irregular ≥ 32 days) modeled as indicator variables. ^d^Wald test of coefficient for body fatness modeled as an ordinal variable. CI, confidence interval; RR, relative risk.
